# Transporting Antitumor Drug Tamoxifen and Its Metabolites, 4-Hydroxytamoxifen and Endoxifen by Chitosan Nanoparticles

**DOI:** 10.1371/journal.pone.0060250

**Published:** 2013-03-20

**Authors:** Daniel Agudelo, Sriwanna Sanyakamdhorn, Shoherh Nafisi, Heidar-Ali Tajmir-Riahi

**Affiliations:** 1 Departement of Chemistry-Biology, University of Québec at Trois-Rivières, Trois-Rivières, Québec, Canada; 2 Department of Chemistry, San Jose State University, San Jose, California, United States of America; Dalhousie University, Canada

## Abstract

Synthetic and natural polymers are often used as drug delivery systems *in vitro* and *in vivo.* Biodegradable chitosan of different sizes were used to encapsulate antitumor drug tamoxifen (Tam) and its metabolites 4-hydroxytamoxifen (4-Hydroxytam) and endoxifen (Endox). The interactions of tamoxifen and its metabolites with chitosan 15, 100 and 200 KD were investigated in aqueous solution, using FTIR, fluorescence spectroscopic methods and molecular modeling. The structural analysis showed that tamoxifen and its metabolites bind chitosan *via* both hydrophilic and hydrophobic contacts with overall binding constants of *K*_tam-ch-15_  = 8.7 (±0.5)×10^3^ M^−1^, *K*_tam-ch-100_  = 5.9 (±0.4)×10^5^ M^−1^, *K*_tam-ch-200_  = 2.4 (±0.4)×10^5^ M^−1^ and *K*_hydroxytam-ch-15_  = 2.6(±0.3)×10^4^ M^−1^, *K*_hydroxytam – ch-100_  = 5.2 (±0.7)×10^6^ M^−1^ and *K*_hydroxytam-ch-200_  = 5.1 (±0.5)×10^5^ M^−1^, *K*_endox-ch-15_  = 4.1 (±0.4)×10^3^ M^−1^, *K*_endox-ch-100_  = 1.2 (±0.3)×10^6^ M^−1^ and *K*_endox-ch-200_  = 4.7 (±0.5)×10^5^ M^−1^ with the number of drug molecules bound per chitosan (*n*) 2.8 to 0.5. The order of binding is ch-100>200>15 KD with stronger complexes formed with 4-hydroxytamoxifen than tamoxifen and endoxifen. The molecular modeling showed the participation of polymer charged NH_2_ residues with drug OH and NH_2_ groups in the drug-polymer adducts. The free binding energies of −3.46 kcal/mol for tamoxifen, −3.54 kcal/mol for 4-hydroxytamoxifen and −3.47 kcal/mol for endoxifen were estimated for these drug-polymer complexes. The results show chitosan 100 KD is stronger carrier for drug delivery than chitosan-15 and chitosan-200 KD.

## Introduction

**Figure 1:**
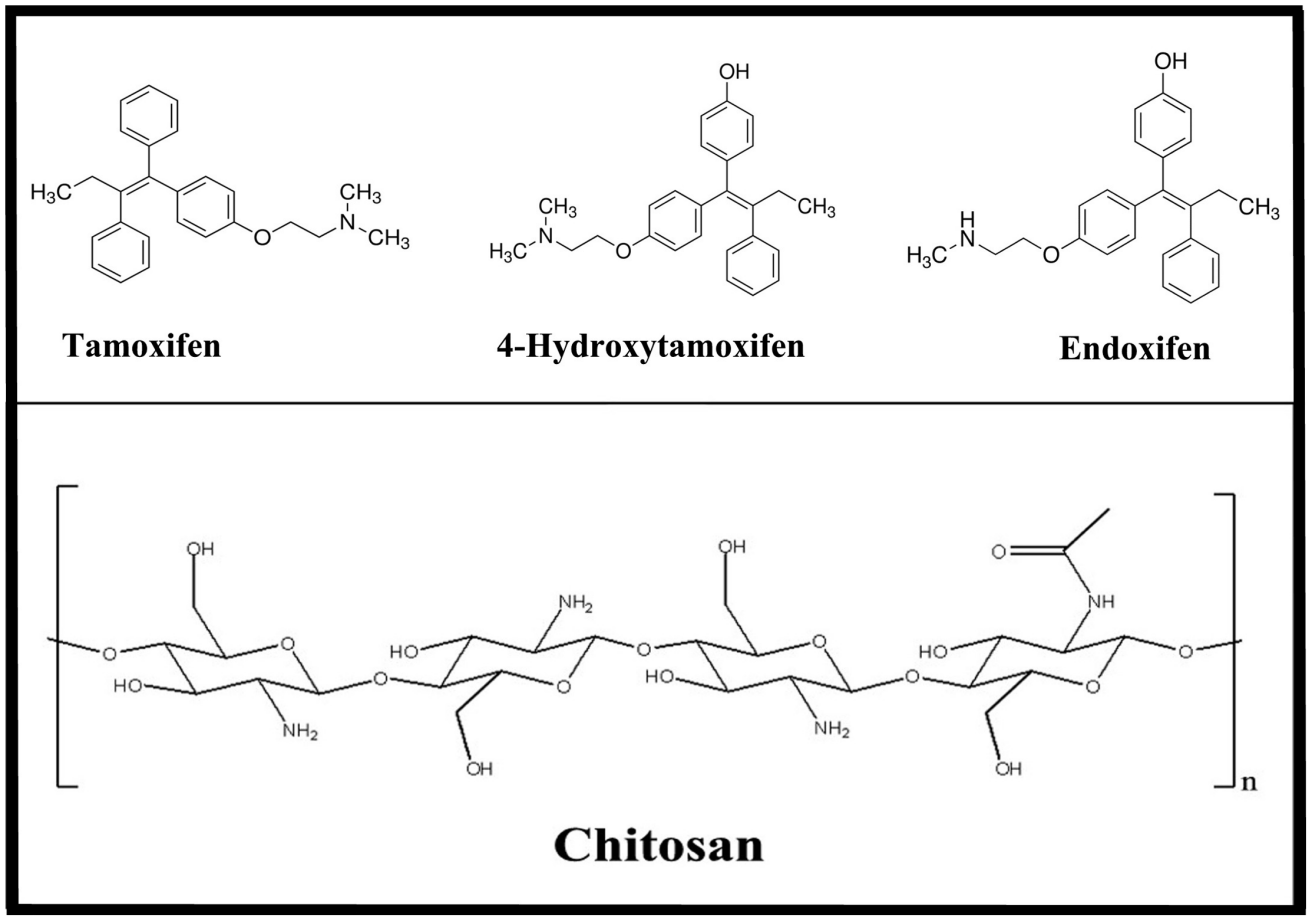
Chemical structures of tamoxifen, 4-hydroxtamoxifen, endoxifen and chitosan.

The nonsteroid antiestrogen tamoxifen [*trans*-1-(4-β-dimethylaminoethoxy-phenyl)-1,2-diphenylbut-1-ene] (Fig. 1) is the most commonly used endocrine treatment for estrogen receptor α (ERα)-positive breast cancer in pre- and post-menopausal women, and it has helped to reduce breast cancer death rate by one third [1]–[3]. It is also used for the prevention of breast cancer in women at high risk of developing the disease [3]. In addition, it is used for the treatment of male breast cancer [4]. Although aromatase inhibitors are currently available for breast cancer treatment in postmenopausal women, tamoxifen is still the “gold standard” of breast cancer therapy because it is cost effective, life saving and is devoid of major adverse side effects in the majority of patients [5], [6]. Tamoxifen is extensively metabolized, and several metabolites have been detected in human serum [7]–[9]. It is metabolized to 4-hydroxytamoxifen and N-desmethyltamoxifen by the action of CYP2D6 and CYP3A4/5 enzymes, respectively. N-desmethyltamoxifen and 4-hydroxy-tamoxifen are further converted to endoxifen (Fig. 1) by the action of CYP2D6 and CYP3A4/5, respectively [10]–[12]. The therapeutic efficacy of tamoxifen is determined by the distribution of the drug into tissues and the availability of the parent drug and its active metabolites in target tissues. However, effective transportation of tamoxifen and its metabolits to target molecules has to be improved and synthetic and natural biopolymers can be used as delivery tools for drug encapsulation.

Chitosan (Fig. 1) is a natural polymer obtained by deacetylation of chitin [13]. It is non-toxic, biocompatible and biodegradable polysaccharide. Chitosan nanoparticles have gained more attention as drug delivery carriers because of their better stability, low toxicity, simple and mild preparation method and providing versatile routes of administration [13]–[17]. The deacetylated chitosan backbone of glucosamine units has a high density of charged amine groups, permitting strong electrostatic interactions with proteins and genes that carry an overall negative charge at neutral pH conditions [13], [14]. The fast expanding research of the useful physicochemical and biological properties of chitosan has led to the recognition of the cationic polysaccharide, as a natural polymer for drug delivery [17]–[20]. Therefore it is of a major interest to study the encapsulation of tamoxifen and its metabolites with chitosan of different sizes, in order to evaluate the efficacy of chitosan nanoparticles in drug delivery.

Fluorescence quenching is considered as a useful and reliable method for measuring binding affinities [21]. Fluorescence quenching is the decrease of the quantum yield of fluorescence from a fluorophore induced by a variety of molecular interactions with quencher molecule [22]. Therefore, it is possible to use quenching of the tamoxifen, 4-hydroxytamoxifen and endoxifen molecules in an attempt to characterize the nature of drug-chitosan interaction. Molecular docking is also an important tool to predict the binding sites of tamoxifen, 4-hydroxytamoifen and endoxifen with chitosan nanoparticles.

The spectroscopic analysis and docking studies of the encapsulation of tamoxifen, 4-hydroxytamoxifen and endoxifen by chitosan nanoparticles 15, 100 and 200 KD in acetate solution at pH 5.5–6.5, using constant polymer concentration and various drug contents are reported. The structural analysis regarding drug binding sites, the effect of chitosan sizes on the stability of drug-polymer complexes and the efficacy of chitosan nanoparticles in drug delivery are discussed here.

## Materials and Methods

### Materials

Purified chitosans 15, 100 and 200 KD (90% deacetylation) were from Polysciences Inc. (Warrington, USA) and used as supplied. Tamoxifen and 4-hydroxytamoxifen were from Sigma Chemical Company. The synthesis of endoxifen was conducted as reported [23]. Other chemicals were of reagent grade and used without further purification.

### Preparation of stock solutions

An appropriate amount of chitosan was dissolved in acetate solution (pH 5.5–6.5). The Drug solutions (1 mM) tamoxifen and its metabolites in ethanol/water (25/75%) were prepared and then diluted by serial dilution in acetate buffer.

### FTIR spectroscopic measurements

Infrared spectra were recorded on a FTIR spectrometer (Impact 420 model, Digilab), equipped with deuterated triglycine sulphate (DTGS) detector and KBr beam splitter, using AgBr windows. Solution of drug was added dropwise to the chitosan solution with constant stirring to ensure the formation of homogeneous solution and to reach the target drug concentrations of 15, 30 and 60 µM with a final chitosan concentration of 60 µM. Spectra were collected after 2 h incubation of chitosan with drug solution at room temperature, using hydrated films. Interferograms were accumulated over the spectral range 4000–600 cm^−1^ with a nominal resolution of 2 cm^−1^ and 100 scans. The difference spectra [(chitosan solution + drug solution) − (chitosan solution)] were generated using free chitosan band around 902 cm^−1^, as standard. This band is related to chitosan ring stretching [20], [24], [25] and does not show alterations upon drug complexation. When producing difference spectra, this band was adjusted to the baseline level, in order to normalize the difference spectra.

### Fluorescence spectroscopy

Fluorimetric experiments were carried out on a Perkin-Elmer LS55 Spectrometer. Stock solution of drug (30 µΜ ) in acetate (pH 5.5–6.5) was also prepared at 24±1°C. Various solutions of chitosan (1 to 200 µM) were prepared from the above stock solutions by successive dilutions at 24±1°C. Samples containing 0.06 ml of the above drug solution and various polymer solutions were mixed to obtain final chitosan concentrations ranging from 1 to 200 µΜ with constant drug content (30 µΜ). The fluorescence spectra were recorded at λ_ex_  = 270–290 nm and λ_em_ from 300 to 450 nm. The intensity of the band at 375 nm from tamoxifen and its metabolites [26] was used to calculate the binding constant (K) according to previous reports [27]–[32].

On the assumption that there are (*n*) substantive binding sites for drug (*Q*) on polymer (*B*), the quenching reaction can be shown as follows:
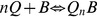
(1)


The binding constant (*K_A_*), can be calculated as:
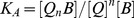
(2)where, [*Q*] and [*B*] are the drug and polymer concentration, respectively, [*Q_n_B*] is the concentration of non fluorescent fluorophore-drug complex and [B_0_] gives total polymer concentration:



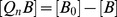
(3)

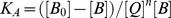
(4)


The fluorescence intensity is proportional to the polymer concentration as described:
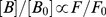
(5)


Results from fluorescence measurements can be used to estimate the binding constant of drug-polymer complex. From eq 4:
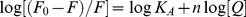
(6)


The accessible fluorophore fraction (*f*) can be calculated by modified Stern-Volmer equation:
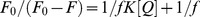
(7)where, *F*_0_ is the initial fluorescence intensity and *F* is the fluorescence intensities in the presence of quenching agent (or interacting molecule). *K* is the Stern-Volmer quenching constant, [Q] is the molar concentration of quencher and *f* is the fraction of accessible fluorophore to a polar quencher, which indicates the fractional fluorescence contribution of the total emission for an interaction with a hydrophobic quencher [21], [22]. The *K* will be calculated from *F_0_/F* =  *K* [Q] +1.

#### Molecular modeling

The docking studies were carried out with ArgusLab 4.0.1 software (Mark A. Thompson, Planaria Software LLC, Seattle, Wa, http://www.arguslab.com). The chitosan structure was obtained from literature report [33] and the drug three dimensional structures were generated from PM3 semi-empirical calculations using Chem3D Ultra 11.0. The whole polymer was selected as a potential binding site, since no prior knowledge of such site was available in the literature (Modeling ref.). The docking runs were performed on the ArgusDock docking engine using regular precision with a maximum of 150 candidate poses. The conformations were ranked using the Ascore scoring function, which estimates the free binding energy. Upon location of the potential binding sites, the docked complex conformations were optimized using a steepest decent algorithm until convergence, with a maximum of 20 iterations. Chitosan donor groups within a distance of 3.5 Å [34] relative to the drug were involved in complex formation.

## Results and Discussion

### FTIR spectra of drug-chitosan complexes

**Figure 2:**
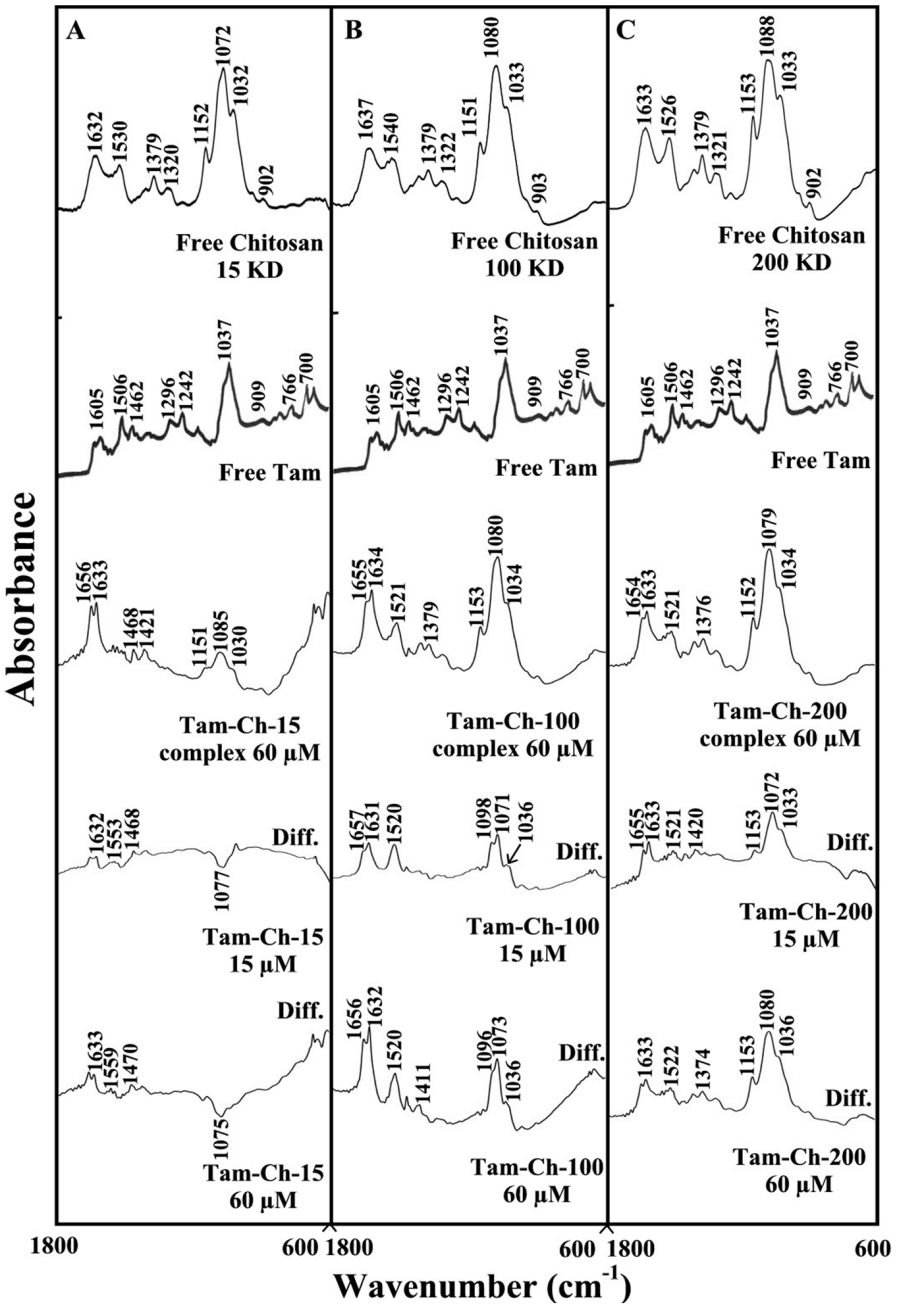
FTIR spectra in the region of 1800–600 cm^−1^ of hydrated films (pH 6) for free chitosan (60 µM) and its tamoxifen complexes for (A) chitosan-15 KD, (B) chitosan-100 KD and (C) chitosan-200 KD with difference spectra (diff.) (bottom two curves) obtained at different drug concentrations (indicated on the figure).

**Figure 3:**
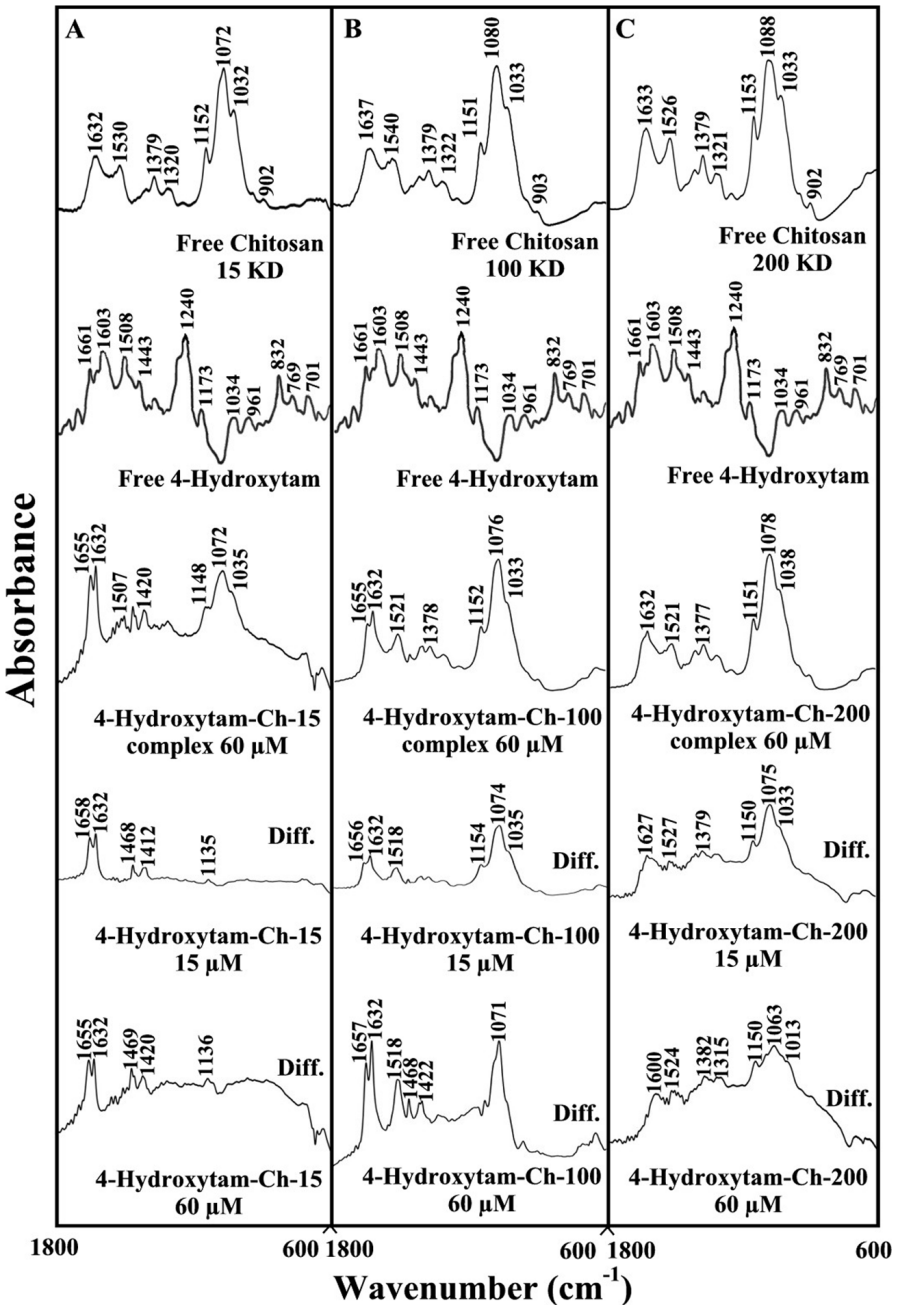
FTIR spectra in the region of 1800–600 cm^−1^ of hydrated films (pH 6) for free chitosan (60 µM) and its 4-hydroxytamoxifen complexes for (A) chitosan-15 KD, (B) chitosan-100 KD and (C) chitosan-200 KD with difference spectra (diff.) (bottom two curves) obtained at different drug concentrations (indicated on the figure).

**Figure 4:**
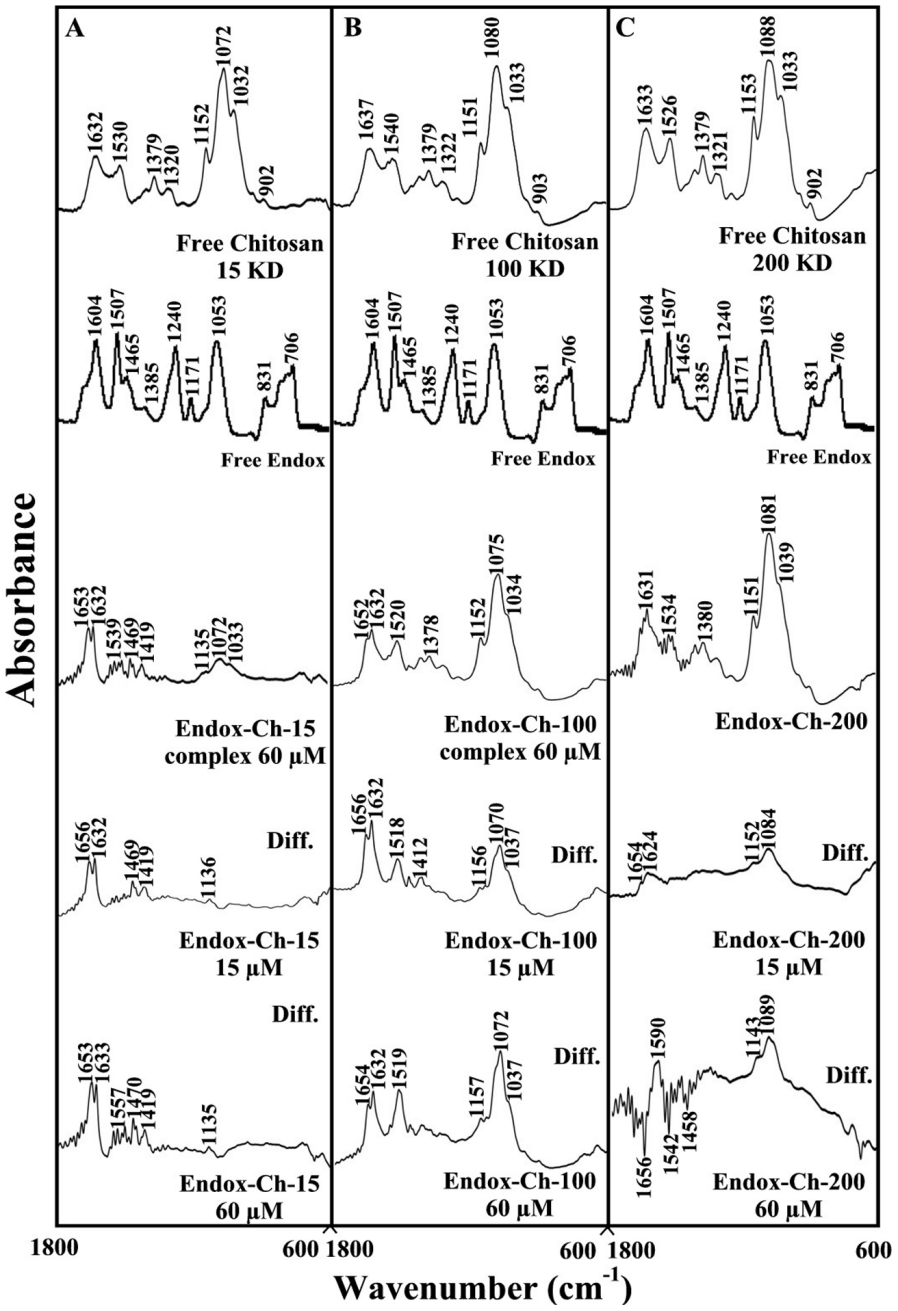
FTIR spectra in the region of 1800–600 cm^−1^ of hydrated films (pH 6) for free chitosan (60 µM) and its endoxifen complexes for (A) chitosan-15 KD, (B) chitosan-100 KD and (C) chitosan-200 KD with difference spectra (diff.) (bottom two curves) obtained at different drug concentrations (indicated on the figure).

The drug-chitosan interactions were characterized by infrared spectroscopy and its derivative methods. The shifting and intensity variations of the chitosan amide I band at 1637–1632 cm^−1^ (mainly C = O stretch) and amide II band at 1540–1526 cm^−1^ (C–N stretching coupled with N-H bending modes) [20], [24], [25] were monitored, upon drug interaction. The difference spectra [(chitosan + drug solution) – (chitosan solution)] were obtained, in order to measure the intensity variations of these vibrations and the results are shown in Figures 2, 3 and 4. Similarly, the infrared spectra of the free chitosan in the region of 3500–2800 cm^−1^ were compared with those of the drug-polymer adducts in Figure 4, in order to examine the drug binding to OH and NH_2_ groups, as well as the presence of hydrophobic contacts in drug-chitosan complexes.

At low drug concentration (15 µM), a minor increase in the intensity was observed for the chitosan amide I at 1637–1632 cm^−1^ and amide II at 1540–1526 cm^−1^, in the difference spectra of the drug-polymer complexes (Figs 2, 3, and 4 diff., 15 µM). The positive features are located at 1633–1625 (tamox-ch-15), 1657–1631 (tamox-ch-100), 1655–1631 (tamox-ch-200), 1658–1632 (4-hydroxtamox-ch-15), 1656–1632 (4-hydroxytamox-ch-100) and 1633–1627 cm^−1^ (4-hydroxytamox-200) and 1656–1632 (endox-ch-15), 1656–1632 (endox-ch-100) and 1654–1624 (endox-ch-200) in the spectra of drug-chitosan complexes (Figs 2, 3 and 4, diff., 15 µM). These positive features are related to the increase in the intensity of the chitosan vibrational frequencies upon drug complexation. The increase in the intensity of the polymer amide I and amide II bands is due to tamoxifen ant its metabolites bindings to polymer C = O, C-N and N-H groups (hydrophilic interaction). Additional evidence to support drug interaction with C-N and N-H groups comes from the shifting of the polymer OH stretching at 3500–3400 cm^−1^ and N-H stretching mode at 3300–3200 cm^−1^, upon drug complexation that will be discussed further on.

As drug concentration increased (30 to 60 µM), a major increase in the intensity of the polymer amide I and amide II vibrations as well as other frequencies was observed with positive features at 1633–1625 (tamox-ch-15), 1656–1632 (tamox-ch-100), 1653–1633 (tamox-ch-200) and 1655-1632 (4-hydroxtamox-ch-15), 1657–1632 (4-hydroxytamox-ch-100) and 1633–1600 cm^−1^ (4-hydroxytamox-200) and 1653–1633 (endox-ch-15), 1654–1632 (endox-ch-100), 1630–1600 cm^−1^ (endox-ch-200) in the spectra of drug-chitosan complexes (Figs 2, 3 and 4, diff., 60 µM). In addition, the polymer amide I and amide II bands exhibited major shifting upon drug complexation (Figs. 2, 3 and 4, complex 60 µM). The major shifting and increase in the intensity of the amide I band in the spectra of the drug-polymer complexes suggests a further interaction of drug with chitosan polar groups.

**Figure 5:**
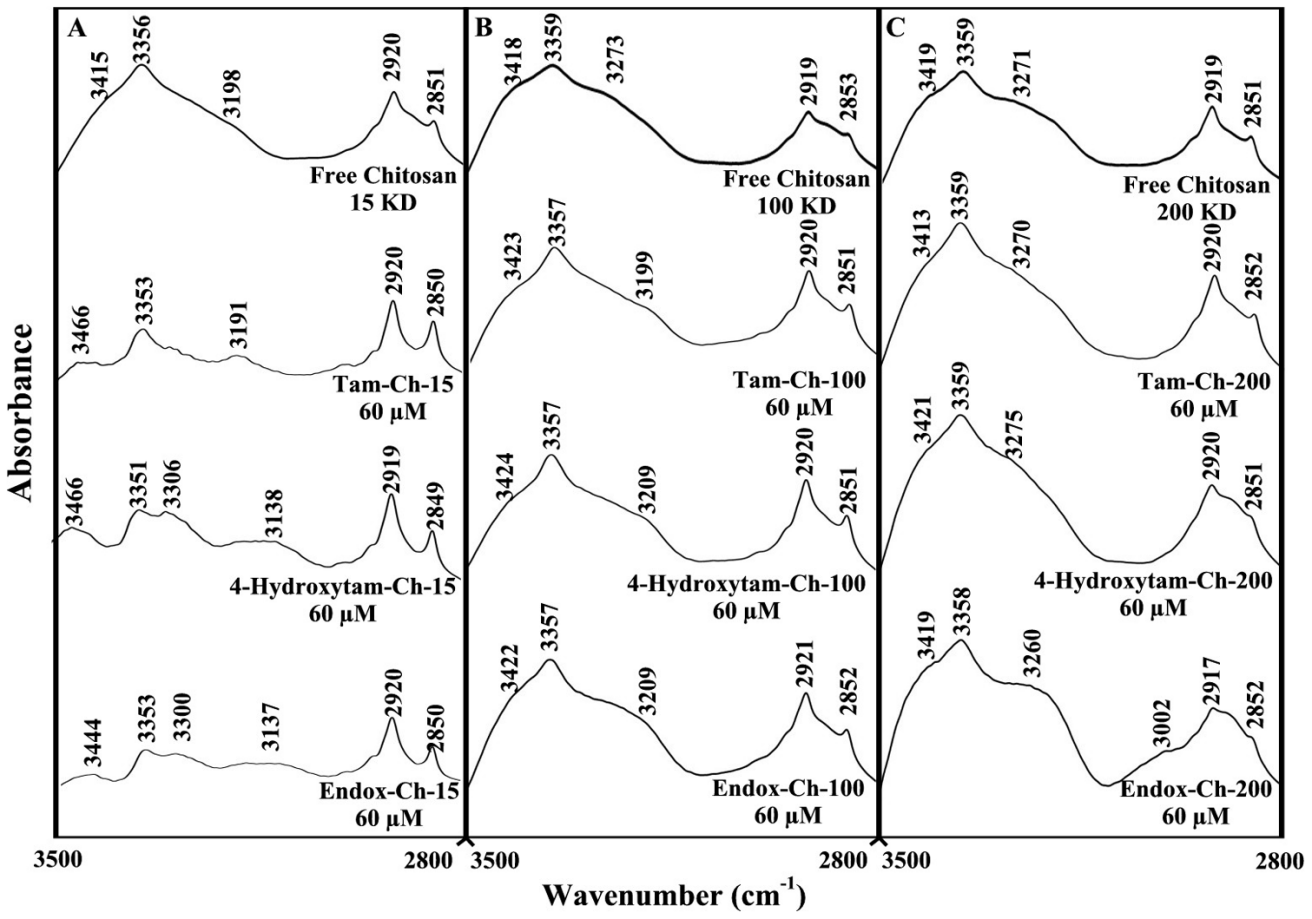
FTIR spectra in the region of 3500-2800 cm^−1^ of hydrated films (pH 6.0) for free chitosan and their tamox, 4-hydroxytamox and endoxifen complexes obtained with 60 µM polymer and 60 **µ M drug concentrations.**

Analysis of the infrared spectra of chitosan in the region of 3500-2800 cm^−1^ showed major shifting of polymer OH, NH and CH stretching modes (Fig. 5) [24], [25]. The polymer OH stretching vibrations at 3415, 3356 ( free ch-15), 3418, 3359 (free ch-100) and 3419, 3359 cm^−1^ (free ch-200) showed major shifting and intensity changes, in the spectra of tamoxifen and its metabolite complexes (Fig. 5). Similarly, the NH stretching vibrations at 3198 (free ch-15), 3273 (free-ch-100) and 3271 cm^−1^ (free ch-200) exhibit major shifting upon drug complexation (Fig. 5). The spectral changes of the polymer OH and NH stretching modes are due to the participation of chitosan OH and NH_2_ group in drug-polymer complexes (hydrophilic contacts). However, the shifting of the polymer symmetric and antisymmetric CH stretching vibrations observed for 2920, 2851 (free ch-15), 2919, 2853 (free ch-100) and 2919, 2851 (free ch-200) in the spectra of tamoxifen, 4-hydroxytamoxifen and endoxifen-polymer complexes is related to the hydrophobic contacts in the drug-chitosan complexes (Fig. 5). The overall spectral changes observed in this region 3500–2800 cm^−1^ are due to the presence of both hydrophilic and hydrophobic contacts, in the drug-chitosan complexes.

### Fluorescence spectra and stability of drug-chitosan complexes

**Figure 6:**
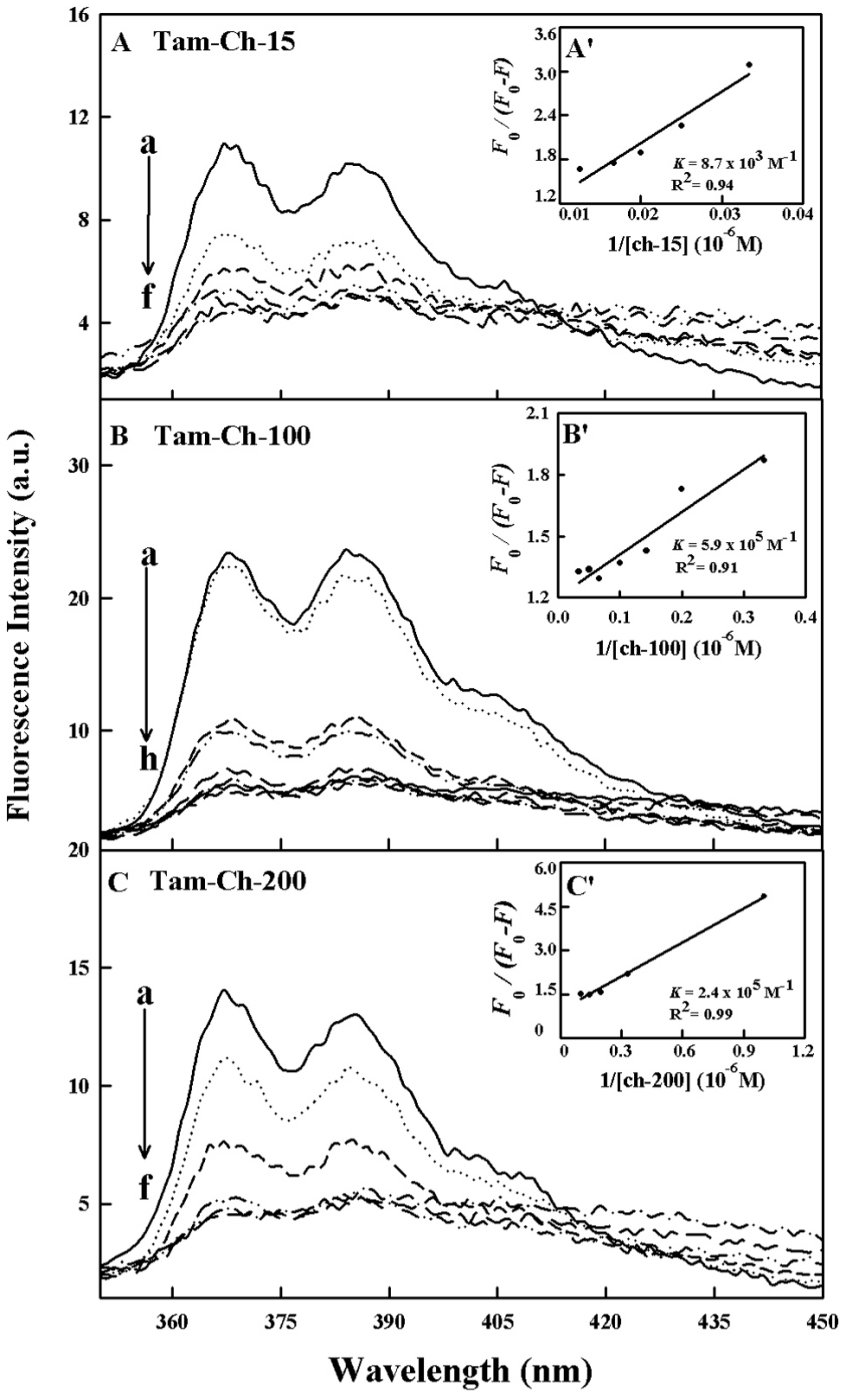
Fluorescence emission spectra of drug-chitosan systems in 10 mM acetate buffer pH 6 at 25°C presented for (A) tam-ch-15: (a) free tam (30 µM), (b-f) with chitosan at 30, 40, 50, 60, 80 and100 µM; (B) tam-chitosan-100: (a) free tam (30 µM), (b–h) chitosan at 1, 3, 5, 7, 10, 15, 20 and 30 µM; (C) tam-ch-200: (a) free tam (30 ) (b-f) with chitosan at 3, 5, 7, 20 and 30, µM; Inset: *K* values calculated by *F*_0_/(*F*_0_ – *F*) vs 1/[chitosan] for A' (tam-chitosan-15), B' (tam- chitosan 100) and C' (tam-chitosan-200).

**Figure 7:**
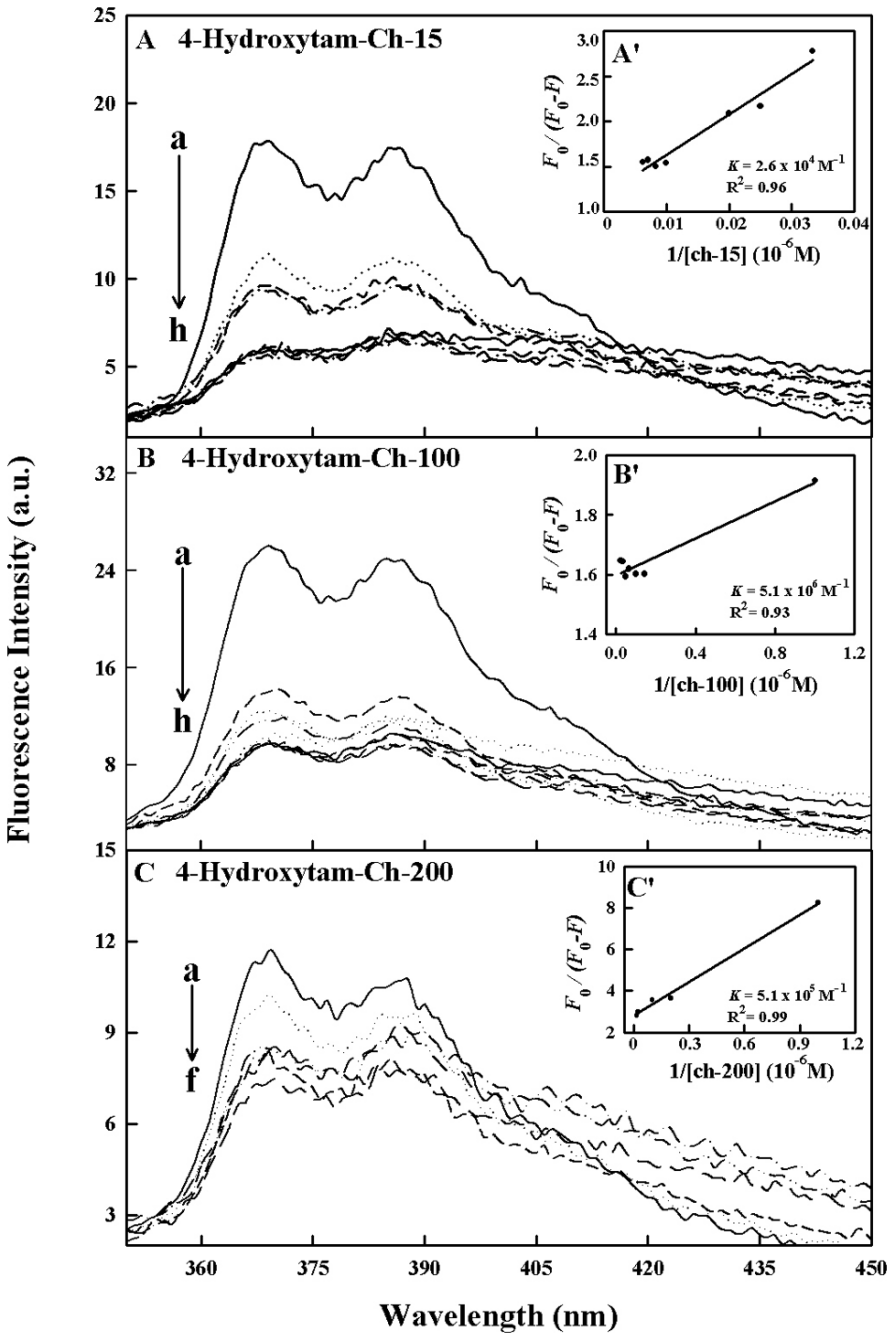
Fluorescence emission spectra of drug-chitosan systems in 10 mM acetate buffer pH 6 at 25°C presented for (A) 4-hydroxytam-ch-15: (a) free 4-hydroxytam (30 µM), (b–h) with chitosan at 30, 40, 50, 60, 80, 100, 120 and 140 µM; (B) 4-hydroxytamx-chitosan-100: (a) free 4.hydroxytam (30 µM), (b–h) chitosan at 1, 3, 5, 7, 10, 15, 20 and 30 µM; (C) 4-hydroxytam-ch-200: (a) free 4-hydroxytam (30 ) (b–f) with chitosan at 3, 5, 7, 20 and 30 µM; Inset: K values calculated by *F*_0_/(*F*_0_ –*F*) vs 1/[chitosan] for A' (4-hydroxytam -chitosan-15), B' (4 hydroxytam – chitosan 100) and C' (4-hydroxytam -chitosan-200).

**Figure 8:**
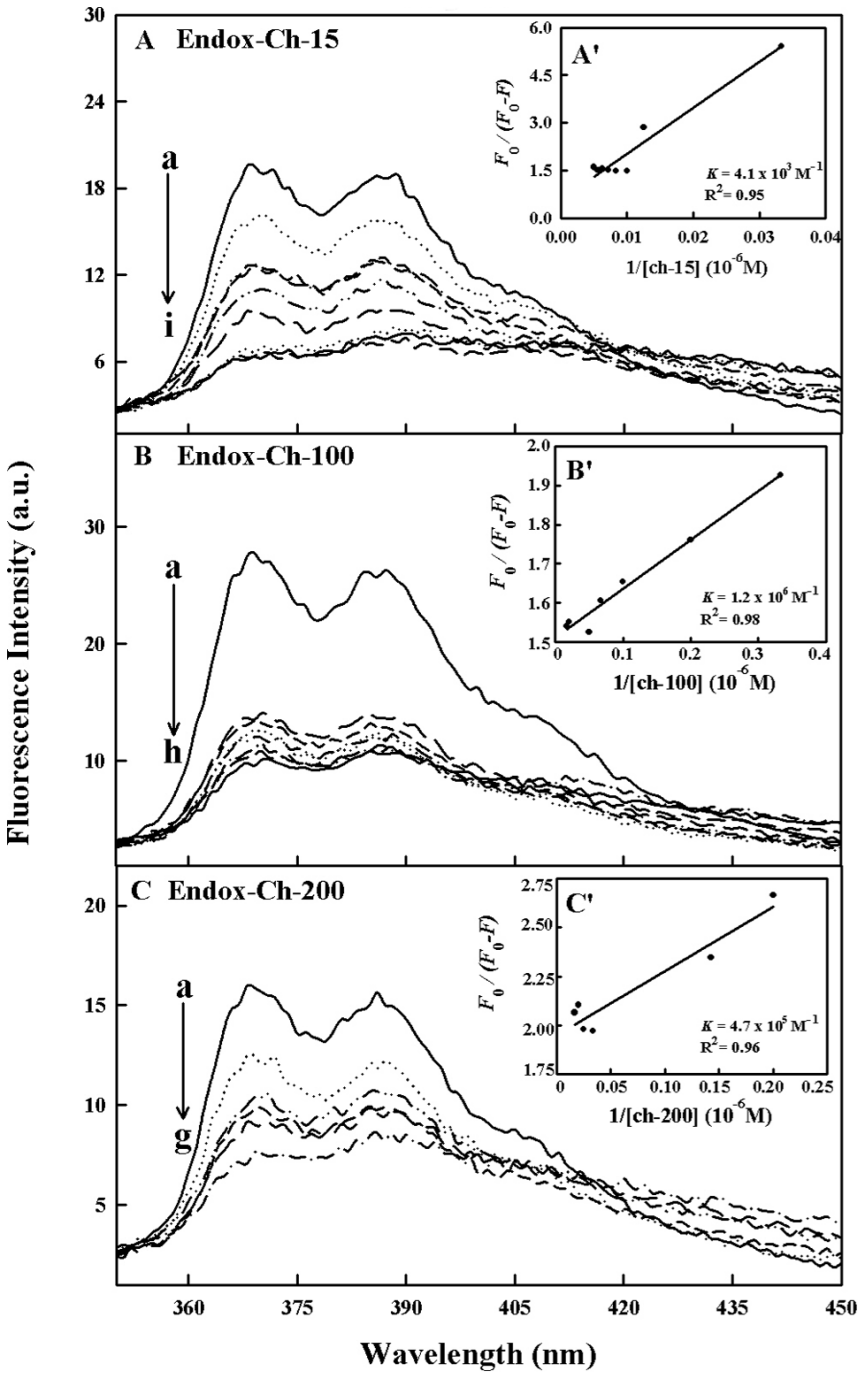
Fluorescence emission spectra of drug-chitosan systems in 10 mM acetate buffer pH 6 at 25°C presented for (A) endox-ch-15: (a) free endox (30 µM), (b-i) with chitosan at 30, 40, 50, 60, 80, 100, 120, 140 and 160 µM; (B) endox-chitosan-100: (a) free endox (30 µM), (b–h) chitosan at 1, 3, 5, 7, 10, 15, 20 and 30 µM; (C) endox-ch-200: (a) free endox (30 ) (b–g) with chitosan at 3, 5, 7, 10, 20 and 30 µM; Inset: *K* values calculated by *F*_0_/(*F*_0_ – *F*) vs 1/[chitosan] for A' (endox-chitosan-15), B' (endox- chitosan 100) and C' (endox-chitosan-200).

Since chitosan is a weak fluorophore, the titrations of tamoxifen, 4-hydroxytamoxifen and endoxifen were done against various polymer concentrations, using drug excitation at 270–290 nm and emission at 350–450 nm [26]. When drug interacts with chitosan, drug fluorescence may change depending on the impact of such interaction on the drug conformation, or *via* direct quenching effect. The decrease of fluorescence intensity of tamoxifen, 4-hydroxytamoxifen or endoxifen has been monitored at 375 nm for drug-chitosan systems (Figs 6-A–C, 7A–C and 8A–C). The plot of *F_0_/(F_0_ – F)* vs 1/[chitosan] is shown in Figs 6A'–C', 7A'–C' and 8A'–C'. Assuming that the observed changes in fluorescence come from the interaction between the drug and chitosan, the quenching constant can be taken as the binding constant of the complex formation. The *K* value given here averages four and six-replicate run for drug-polymer systems. Each run involves several different concentrations of chitosan (Figs 6A–C, 7A–C and 8A–C). The overall binding constants were *K*_tam-ch-15_  = 8.7 (±0.5)×10^3^ M^−1^, *K*_tam-ch-100_  = 5.9 (±0.4)×10^5^ M^−1^, *K*_tam-ch-200_  = 2.4 (±0.4)×10^5^ M^−1^ and *K*_hydroxytam-ch-15_  = 2.6 (±0.3)×10^4^ M^−1^, *K*_hydroxytam – ch-100_  = 5.2 (±0.7)×10^6^ M^−1^ and *K*_hydroxytam-ch-200_  = 5.1 (±0.5)×10^5^ M^−1^, *K*_endox-ch-15_  = 4.1 (±0.4)×10^3^ M^−1^, *K*_endox-ch-100_  =  1.2 (±0.3)×10^6^ M^−1^ and *K*_endox-ch-200_  = 4.7 (±0.5)×10^5^ M^−1^ (Figs 6A'–C',7A'–C' and 8A'–C' and Table 1). The order of binding constants calculated for the drug-chitosan adducts, showed ch-100>200>15 KD with more stable complexes formed with 4-hydroxytamoxifen than tamoxifen and endoxifen (Table 1). It is important to note that ch-15 is smaller than ch-100 and ch-200, while drug-interaction is mainly via positively charged chitosan NH_2_ groups, as polymer size gets larger the increases in overall polymer charges will result in stronger drug-polymer complexation. However, in the case of ch-200 aggregation of polymer occurs at pH near 6, which leads to lesser affinity of the aggregated polymer for drug interaction (self-aggregation is less observed for ch-15 and ch-100). Therefore, ch-100 forms more stable complexes than the ch-15 and ch-200 (Table 1). On the other hand, 4-hydroxytamoxifen forms more stable complexes than tamoxifen and endoxifen, due to more hydrophilic and hydrophobic characters of 4-hydroxytamoxifen than those of other analogues (Table 1). The *f* value calculated from Eq. 7 represents the mole fraction of the accessible population of fluorophore to quencher. The *f* values were from 0.2 to 0.65 for these drug-chitosan complexes indicating a large portion of fluorophore was exposed to quencher.

**Table 1:** Binding parameters for drug-chitosan complexes.

Complexes	*K*_sv_ (M^−1^)			*K_a_ (M^−1^)*			*n*			*K_q_ (M^−1^s^−1^)*		
	Ch-15	Ch-100	Ch-200	Ch-15	Ch-100	Ch-200	Ch-15	Ch-100	Ch-200	Ch-15	Ch-100	Ch-200
Tamox	6.8×10^7^	5.4×10^6^	8.5×10^7^	8.7×10^3^	5.9×10^5^	2.4×10^5^	1.5	2.8	0.9	3.2×10^16^	2.6×10^15^	4.0×10^16^
Hydroxytam	1.8×10^8^	6.3×10^7^	4.4×10^8^	2.6×10^4^	5.1×10^6^	5.1×10^5^	1.3	0.6	0.7	8.5×10^16^	3.0×10^16^	2.1×10^17^
Endox	5.8×10^7^	5.2×10^7^	1.7×10^8^	4.1×10^3^	1.2×10^6^	4.7×10^5^	1.2	0.5	0.5	2.7×10^16^	2.4×10^16^	7.9×10^16^

**Figure 9:**
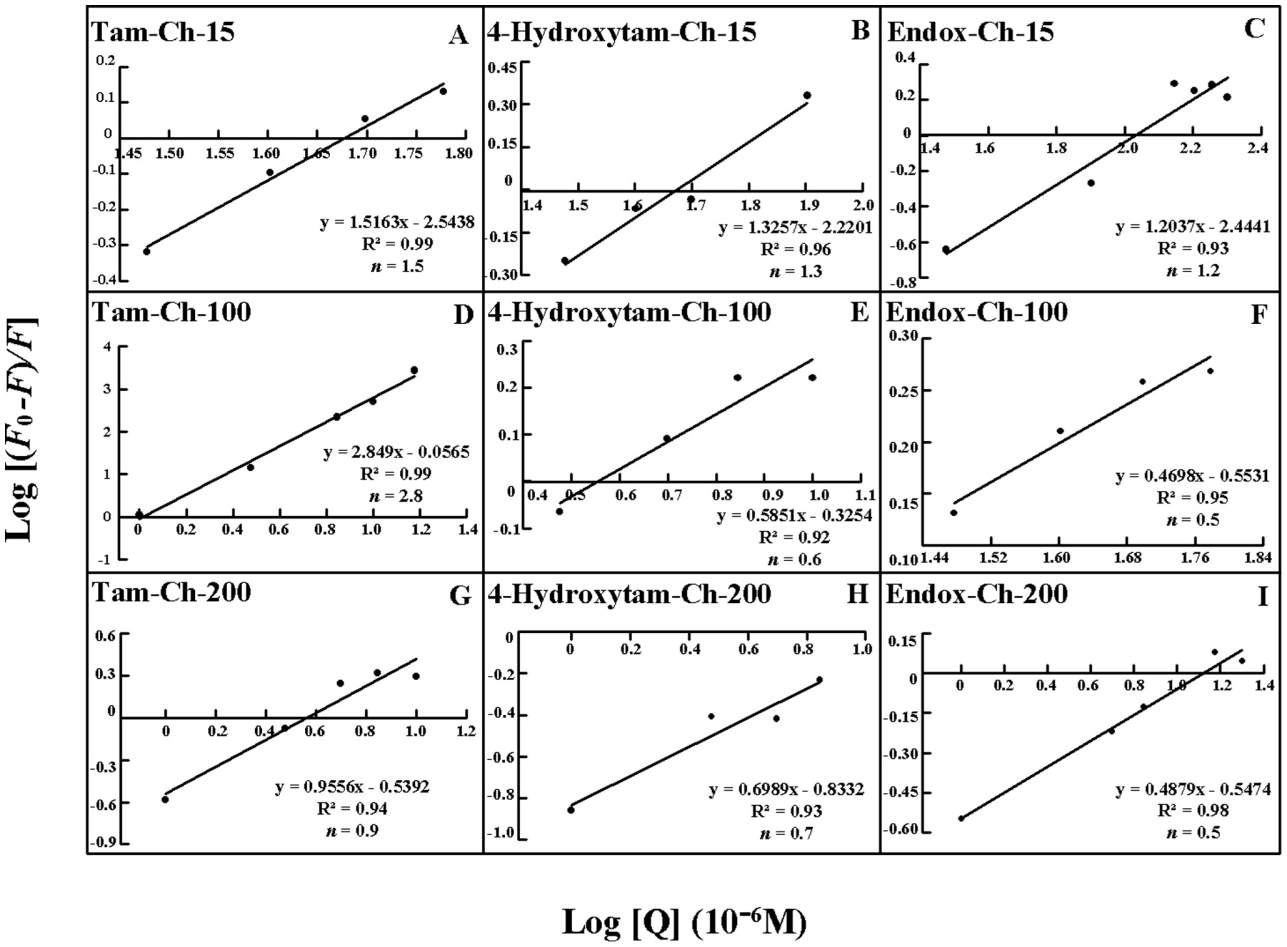
Stern-Volmer plots of fluorescence quenching constant (*K_Q_*) for the chitosans and their drug complexes at different chitosan concentrations.

The number of drug molecules bound per polymer (*n*) is calculated from log [(*F0 -F*)/*F*]  =  log*KS* + n log [chitosan] for the static quenching [20], [35]–[38]. The *n* values from the slope of the straight line plot showed 0.5 to 2.8 drug molecules that are bound per chitosan molecule (Fig. 9 and Table 1). The results indicate some degree of cooperativity for drug-polymer interaction.

**Figure 10:**
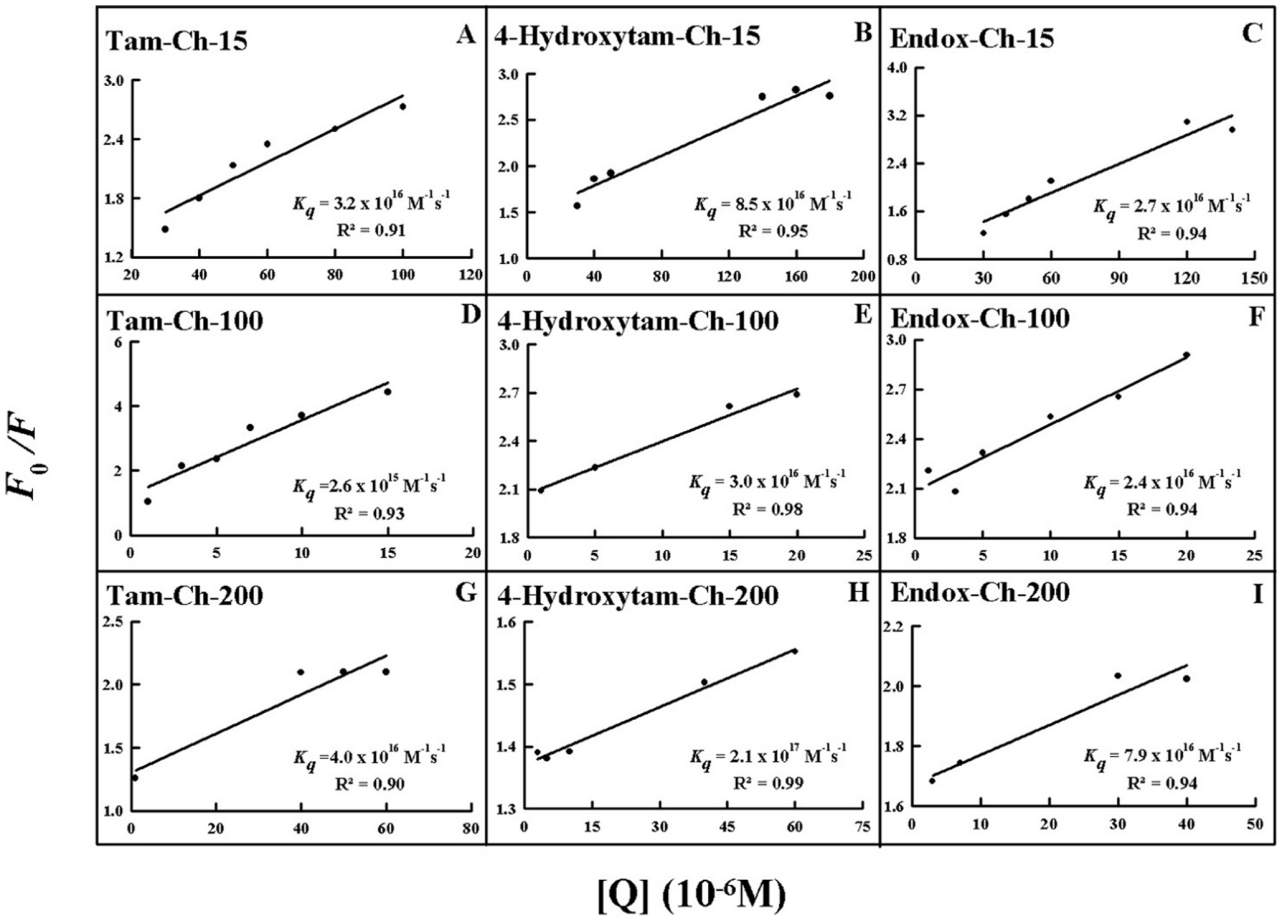
The plot of Log (*F_0_-F*)/*F* as a function of Log (chitosan concentrations) for the number of bound drug molecules per chitosan (*n*) for drug-polymer complexes.

In order to verify the presence of static or dynamic quenching in drug-chitosan complexes we have plotted *F_0_/F* against *Q* to estimate the quenching constant (*KQ*) and the results are shown in Fig. 10. The plot of *F_0_/F* versus Q is a straight line for drug*-*chitosan adducts indicating that the quenching is mainly static in these drug-polymer complexes (Fig. 10). The quenching constant *K*_Q_ was estimated according to the Stern-Volmer equation:
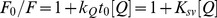
(8)where *F_0_* and *F* are the fluorescence intensities in the absence and presence of quencher, [Q] is the quencher concentration and *K*_sv_ is the Stern-Volmer quenching constant [39] which can be written as *K*_sv_  =  k_Q_t_0_; where *k_Q_* is the bimolecular quenching rate constant and t_0_ is the lifetime of the fluorophore in the absence of quencher about 2.1 ns for free tamoxifen around neutral pH [26], [40]. The quenching constants (*K_Q_*) are 3.2×10^16^ M^−1^/s for tamox*-*ch-15, 2.6× M^−1^/s for tamox-ch-100, 4.0×10^16^M^−1^/s for tamox*-*ch-200 and 8.5×10^16^M^−1^/s for 4-hydroxytamox-ch-15, 3.0×10^16^ M^−1^/s for 4-hydroxytamox*-*ch-100, 2.1×10^17^M^−1^/s for 4-hydroxytamox-ch-200 and 2.7×10^16^ M^−1^/s for endox*-*ch-15, 2.4×10^16^ M^−1^/s for endox-ch-100 and, 7.9×10^16^M^−1^/s for endox*-*ch-200 (Fig. 10 and Table 1). Since these values are much greater than the maximum collisional quenching constant (2.0×10^10^M^−1^/s), the static quenching is dominant in these drug-polymer complexes [39].

### Docking

**Figure 11:**
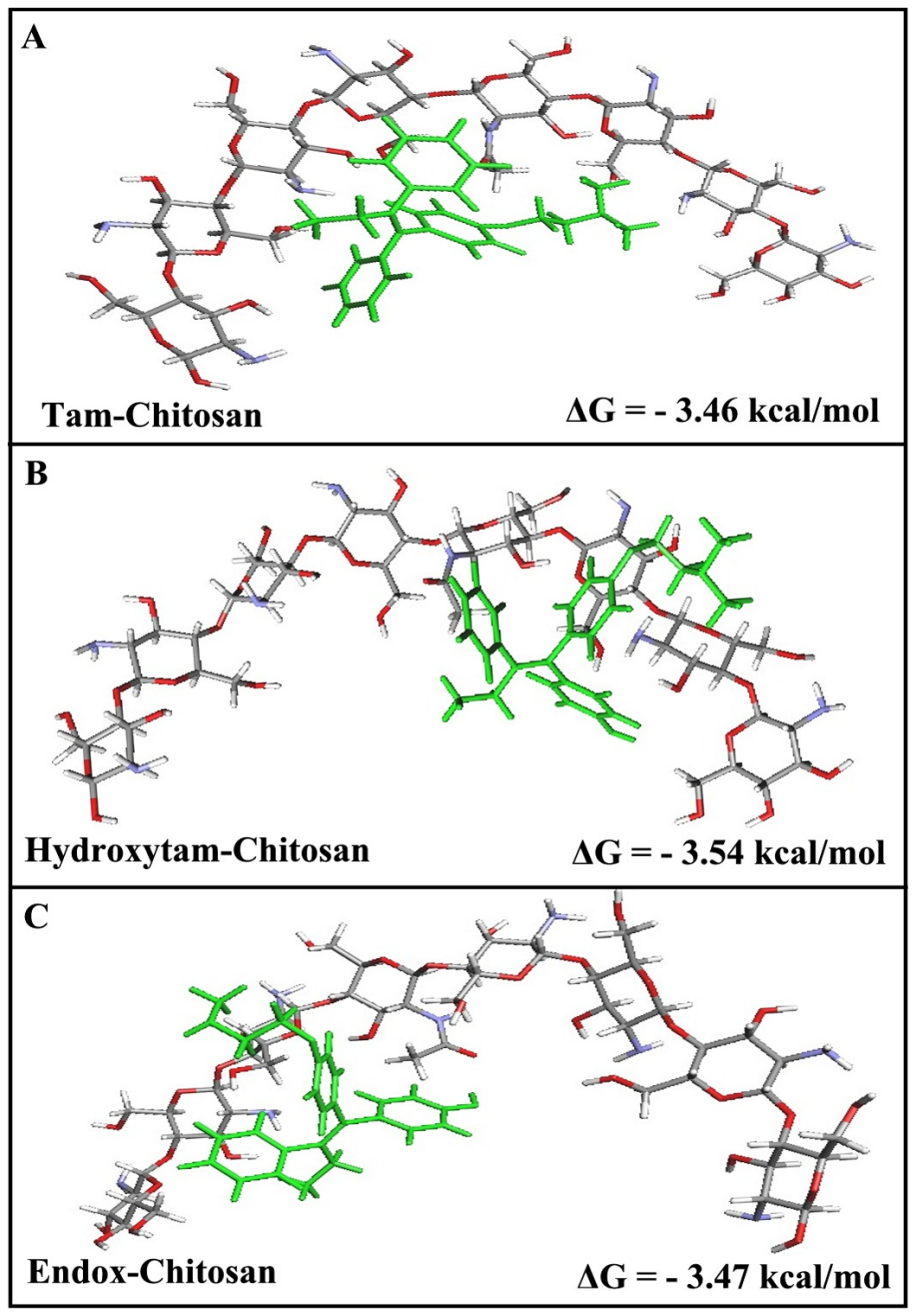
Best docked conformations of drug–chitosan complexes. (**A**) for tamoxifen bound to chitosan and (**B**) for 4-hydroxytamoxifen bound to chitosan (**C**) for endoxifen bound to chitosan with free binding energy.

The spectroscopic data were combined with docking experiments in which tamoxifen, 4-hydroxytamoxifen and endoxifen molecules were docked to chitosan to determine the preferred binding sites on the chitosan. The models of the docking for drug are shown in Fig. 11. The docking results showed that tamoxifen and its metabolites are surrounded by several donor atoms of chitosan residue on the surface with a free binding energy of −3.46 kcal/mol for tamox-chitosan −3.54 kcal/mol for 4-hydroxytamoxifen-chitosan and −3.47 kcal/mol for endoxifen-chitosan complexes (Fig. 11). It is evident that tamoxifen and its metabolites are not surrounded by similar donor groups showing different binding modes, in these drug-chitosan complexes with more stable adducts formed for 4-hydroxytamoxifen, which is consistent with our spectroscopic results (Fig 11 and Table 1).

### Conclusion

The spectroscopic and docking results presented here show that tamoxifen and its metabolites bind chitosan *via* different binding modes. Major hydrophobic and hydrophilic interactions *via* chitosan charged NH_2_ groups are observed in these drug-polymer complexes. The order of drug-chitosan binding is ch-100>ch-200>ch-15. Stronger complexes formed for 4-hydroxytamoxifen due to more hydrophilic and hydrophobic characters. Chitosan-100 KD is a stronger carrier for tamoxifen and its metabolites than chitosan 15 and chitosan 100 KD for drug delivery *in vitro*.
